# Entropy Measures in Analysis of Head up Tilt Test Outcome for Diagnosing Vasovagal Syncope

**DOI:** 10.3390/e20120976

**Published:** 2018-12-16

**Authors:** Katarzyna Buszko, Agnieszka Piątkowska, Edward Koźluk, Tomasz Fabiszak, Grzegorz Opolski

**Affiliations:** 1 Department of Theoretical Foundations of Bio-Medical Science and Medical Informatics, Collegium Medicum, Nicolaus Copernicus University, 85-067 Bydgoszcz, Poland; 2 Department of Emergency Medicine, Wroclaw Medical University, 02-091 Wroclaw, Poland; 3 1st Department of Cardiology, Medical University of Warsaw, 02-091 Warsaw, Poland; 4 Department of Cardiology and Internal Diseases, Collegium Medicum, Nicolaus Copernicus University, 85-067 Bydgoszcz, Poland

**Keywords:** sample entropy, permutation entropy, fuzzy entropy, Shannon entropy, conditional entropy, vasovagal syndrome, head up tilt test

## Abstract

The paper presents possible applications of entropy measures in analysis of biosignals recorded during head up tilt testing (HUTT) in patients with suspected vasovagal syndrome. The study group comprised 80 patients who developed syncope during HUTT (57 in the passive phase of the test (HUTT(+) group) and 23 who had negative result of passive phase and developed syncope after provocation with nitroglycerine (HUTT(−) group)). The paper focuses on assessment of monitored signals’ complexity (heart rate expressed as R-R intervals (RRI), blood pressure (sBP, dBP) and stroke volume (SV)) using various types of entropy measures (Sample Entropy (SE), Fuzzy Entropy (FE), Shannon Entropy (Sh), Conditional Entropy (CE), Permutation Entropy (PE)). Assessment of the complexity of signals in supine position indicated presence of significant differences between HUTT(+) versus HUTT(−) patients only for Conditional Entropy (CE(RRI)). Values of CE(RRI) higher than 0.7 indicate likelihood of a positive result of HUTT already at the passive phase. During tilting, in the pre-syncope phase, significant differences were found for: (SE(sBP), SE(dBP), FE(RRI), FE(sBP), FE(dBP), FE(SV), Sh(sBP), Sh(SV), CE(sBP), CE(dBP)). HUTT(+) patients demonstrated significant changes in signals’ complexity more frequently than HUTT(−) patients. When comparing entropy measurements done in the supine position with those during tilting, SV assessed in HUTT(+) patients was the only parameter for which all tested measures of entropy (SE(SV), FE(SV), Sh(SV), CE(SV), PE(SV)) showed significant differences.

## 1. Introduction

Entropy measures are widely used for assessment of biosignals’ irregularity and complexity [1,2,3,4,5,6,7,8,9,10,11,12,13,14,15,16,17,18,19,20,21,22,23,24]. The computation of entropy based on the first fundamental definitions needed a large number of data to achieve convergence. The entropy measure proposed by Pincus in 1991, which is called Approximate Entropy allowed calculating the entropy of finite and noisy data [5,8,25]. Since that time many entropy measures have been proposed and the family of Approximate Entropy expanded by its improvements, i.e., Sample Entropy, Fuzzy Entropy, Multiscale Entropy etc. Sample Entropy proposed in 2000 by Richmann and Moorman [4] is used in clinical practice [26]. The most analyzed signals are R-R interval (RRI) recordings extracted from electrocardiogram (ECG). The changes in irregularity and complexity of RRI assessed by entropy were investigated for heart failure, dysfunctions of autonomic nervous system and also in assessment of HRV complexity in healthy subjects tested in controlled interventions like e.g., regular walking [14] or head up tilt test (HUTT) [1,16,26,27,28,29].

As a part of our research, we also investigated the complexity and irregularity of biosignals measured during head up tilt testing carried out in patients suffering from unexplained syncope. According to The European Society of Cardiology, syncope is a transient loss of consciousness (TLOC) due to cerebral hypoperfusion characterized by a rapid onset, short duration, and spontaneous complete recovery [30]. Syncope usually results in a fall. The main types of syncope are: cardiac, orthostatic and neuro-cardiogenic [31]. The analyzed group of patients suffered from vasovagal syncope, which belongs to the neuro-cardiogenic type. The vasovagal syncope usually appears following strong stress or emotions. Some patients tend to fall during prolonged upright tilt, especially in stuffy rooms, another fall e.g., during the vein puncture [31].

The pathophysiological mechanism and principal causes of this type of syncope are not fully known. In diagnosing of syncope the primary point is a history taken from the patient with a particular focus on present and previous episodes [30]. The second step is a physical examination that includes HUTT, usually performed with Task Force Monitor (TFM) [32,33,34]. The test consists of lying on a table that can tilt to different angles (60 to 90-degree angle), followed by a sudden sudden tilt to upright position and prolonged standing until syncope occurs. During the test three basic signals are recorded: electrocardiogram (ECG), systolic blood pressure (sBP) and diastolic blood pressure (dBP) and sometimes additionally impedance cardiography (ICG). Although according to the 2018 ESC Guidelines for the diagnosis and management of syncope, the HUTT has acceptable specificity and sensitivity in vasovagal syndrome (VVS) diagnosis [30], it has not been accepted as the gold standard diagnostic procedure for vasovagal syncope. Therefore, there is no single recommended protocol of HUTT. The most commonly used protocols are the Westminster and the Italian ones. The protocols differ in the initial stabilization phase, duration, tilt angle, type of support, and pharmacological provocation.

The aim of this paper is to evaluate if entropy of simultaneously measured RRI, sBP, dBP and SV can be a useful tool for prediction of vasovagal syndrome during HUTT. The optimal predictive tool should be capable of discriminating, already at the introductory supine phase, between the patients who will develop syncope during the passive tilt phase of the test and those who will not. However, a measure allowing early post-tilt prediction of the passive phase outcome soon after the onset of tilting would also be very useful. It would allow reduction of the tilt test duration by early skipping to the provocation phase once the passive phase is deemed to be non-diagnostic. Therefore, in our research we also attempted to identify which entropy measures meet these requirements. Having various entropy measures under consideration, we chose five: Sample Entropy (SE), Fuzzy Entropy (FE), Shannon Entropy (ShE), Conditional Entropy (CE) and Permutation Entropy (PE). The main reason of such choosing were various properties of the measures in terms of data length and the presence of noise. Shannon Entropy was chosen from the historical reasons, as it is the best known classical entropy measure. Sample Entropy and Fuzzy Entropy represent the family of Approximate Entropy and have the same mathematical background. They were widely used in analysis of cardiovascular signals. In turn, the Conditional Entropy has another mathematical philosophy and it has good properties in the analysis of the short data sets. The Permutation Entropy works well in presence of noise and it is useful in analyzing of non-stationary data.

The paper is arranged in the following order: Section 2—brief description of the entropy measures, Section 3—materials and methods, Section 4—results of the research, Section 5—discussion.

## 2. Entropy Measures

The concept of entropy has a long history, which began with investigations in thermodynamics. In 1867 Rudolf Clausius proposed entropy as a measure of system uncertainty [35]. Since that time many formulas of entropy have been introduced and many concepts were elaborated based on this idea. The main inspiration for the development of the idea of entropy was its implementation in the information theory and time series analysis. In our investigation we applied various entropy measures: Sample Entropy (SE), Fuzzy Entropy (FE), Shannon Entropy (ShE), Conditional Entropy (CE) and Permutation Entropy (PE) to analyze biosignals. The theoretical foundations of the mentioned entropies and algorithms for their calculations have been widely described in the literature [4,5,8,25,36,37,38,39]. In this section, we briefly present the measures of entropy mentioned above and their properties.

### 2.1. Sample Entropy

Sample Entropy (SE) was introduced by Moorman and Richmann in 2000 [4]. Their formula of entropy was inspired by an ealier calculation of entropy proposed by Pincus and widely known as Approximate Entropy (ApEn) [25]. The idea of both entropies is based on measuring the probability that similar sequences of points in time series remain similar for increment sequences [25]. Due to self-matches ApEn is a biased estimator and consequently its values depend on time series length and a loss of relative consistency [4,5]. Despite these limitations, ApEn has extensive applications in cardiovascular physiology [1,2,3,4,5,6,7,8,9,10,40]. Sample Entropy does not have such limitations and has better relative consistency in application for short data sets.

The formula of SE for time series ${\left\{x_{i}\right\}}_{i=1}^{N}=\left[x_{1},x_{2},x_{3},\ldots ,x_{N}\right]$ of length *N*, embedding dimension *m* and similarity threshold *r* is as follows:

(1) $$SE\left(m,\;r,N\right)=-ln\left(\frac{\sum_{i=1}^{N-m}A_{i}^{m+1}}{\sum_{i=1}^{N-m}A_{i}^{m}}\right)$$

where $A_{i}^{m}\left(r\right)$ is the number of vectors that are similar according to similarity criterion:

(2) $$d_{i,j}^{m}\leq r\;,\;\mathrm{where}\;1\leq i,\;j\leq N-m\;\mathrm{and}\;i\neq j$$

and

(3) $$d_{i,j}^{m}\leq r\;,\mathrm{where}\;1\leq i,\;j\leq N-m\;\mathrm{and}\;i\neq j$$

Correspondingly $A_{i}^{m+1}\left(r\right)$ is the number of vectors that are similar according to similarity criterion for m+1 and fulfill the criterion $\left(d_{i,j}^{m+1}\leq r\right)$. The embedding dimension *m* and similarity threshold *r* are chosen arbitrarily and for cardiovascular signals *m* is usually set as 2 and r=0.2×SD, where SD stands for standard deviation of $x_{i}$. The differences between ApEn and SE are clearly visible. In fact, for both entropies the decision ruling for the vector similarity is the same. This rule is based on the Heaviside function:

(4) $$H\left(d_{i,j}^{m},r\right)=\{\begin{array}{cc} 1 & d_{i,j}^{m}\leq r\\ 0 & d_{i,j}^{m}>r \end{array}$$

Such a rigorous rule results in statistical instability of the entropy values, because they can change abruptly when the threshold *r* changes slightly [37,41]. That problem was resolved by the introduction of Fuzzy Entropy described in the next subsection.

### 2.2. Fuzzy Entropy

The main idea of Fuzzy Entropy (FE) is the same as in the case of SE and ApEn. However, there is a difference in membership function, where the Heaviside function is replaced by function [26,41]:

(5) $$M\left(d_{i,j}^{m},r\right)=e^{-ln2{\left(d_{i,j}^{m}/r\right)}^{2}}$$

Thus, in the entropy calculation (1) one has $A_{i}^{m}=\overset{N-m}{\underset{j=1,j\neq i}{\sum }}e^{-ln2{\left(d_{i,j}^{m}/r\right)}^{2}}$ and corresponding changes in the formula for $A_{i}^{m+1}$. The calculation of the distance $d_{i,j}^{m}$ in similarity criterion is also slightly different, i.e.,

(6) $$d_{i,j}^{m}=max\left(\left|\left[x_{i+k}-{\bar{x}}_{i}\right]-[x_{j+k}-{\bar{x}}_{j}]\right|\right),\;0\leq k\leq m-1$$

where ${\bar{x}}_{i}$ and ${\bar{x}}_{j}$ are local means of the compared vectors. The introduced fuzzy membership function (5) that substituted the Heaviside function (4), changes the rigid boundary effect into smooth boundary effect of the threshold *r*. In FE calculation the similarity between vectors is evaluated based on their shape and in consequence it has better consistency than SE [26,37,41]. Nonetheless, in both algorithms the calculations are based on embedding dimension *m*, threshold *r* and length of the signal *N*. In the analysis of cardiovascular signals there are usually set as: *m* = 2 and r=0.2×SD [41].

### 2.3. Shannon Entropy

Shannon Entropy (ShE) is a wildly known, fundamental mathematical formula describing the uncertainty and loss of information in dynamical systems. It was proposed in 1948 by Claude Shannon [36] as function:

(7) $$H=-K\overset{k}{\underset{i=1}{\sum }}p\left(i\right)logp\left(i\right)$$

where *K* is a constant connected with the unit of measurements and p(i) is a probability of a system being in i. The ShE is a much wider concept than the entropy proposed by Clausius [35] because it is applied in general to quantities described only by a probability distribution. This idea of entropy was developed by many researches and applied in many scientific fields.

### 2.4. Conditional Entropy

In information theory Conditional entropy (CE) evaluates the entropy of random variable *Y* conditioned on random variable *X* (H(Y|X)). It evaluates the information needed to obtain the variable *Y* with the known variable *X*. In the time series analysis, the CE quantifies the information carried by the sampling point of length *m* + 1 with the known previous sampling point of length *m*. The information is assessed with the ShE. The theoretical background of CE and algorithm for its calculations were described in [14,38]. Here, we briefly go over the final form of the pattern for CE calculation.

Let us take the time series ${\left\{x_{i}\right\}}_{i=1}^{N}=\left[x_{1},x_{2},x_{3},\ldots .x_{N}\right]$ of length *N*, embedding dimension *m* and time delay  τ. The CE(m,τ) is defined by [14]:

(8) $$CE\left(m,\tau \right)=ShE\left(z_{j}\right)-ShE\left(w_{i}\right)+perc\left(m\right)ShE\left(1\right)$$

The time series ${\left\{x_{i}\right\}}_{i=1}^{N}$ are quantized with the quantization levels ξ of amplitude $\varepsilon =\left(x_{max}-x_{min})/\zeta \right)$. $ShE\left(z_{j}\right)$ and $ShE\left(w_{i}\right)$ are the Shannon Entropy calculated for $z_{j}$ and $w_{i}$, which are corresponding to the state space vectors codified in decimal format [14,38]. The term perc(m) means the percentage of $w_{i}$, that occurred only once in the data and ShE(1) is the Shannon Entropy for the series ${\left\{x_{i}\right\}}_{i=1}^{N}$ after quantization. In our analysis following by [14,38] we have taken: m=2, τ=1 and ξ=6.

### 2.5. Permutation Entropy

The Permutation Entropy (PE) was proposed by Brandt et al. in [39] and it is based on the assessment of diversity of ordinal patterns in time series ${\left\{x_{i}\right\}}_{i=1}^{N}$. The main procedure for calculation of PE is creation of $\pi_{j}$ vectors (1≤j≤m!) by resorting the state–space vectors in increasing order and calculating the frequency $\left(p_{j}\left(m,\;\tau \right)\right)$ of each $\pi_{j}$. The formula for PE calculation is as follows [14,39]:

(9) $$PE\left(m,\tau \right)=-\;\frac{1}{{log}_{2}m!}\sum_{j=1}^{m!}p_{j}\left(m,\tau \right){log}_{2}[p_{j}\left(m,\tau \right)]$$

where *m* is embedding dimension and τ is a time delay. For calculations presented in this paper we assumed that *m* = 2 and τ=1.

## 3. Materials and Methods

### 3.1. Study Group

This is a retrospective, single-center analysis of data collected between year 2005 and 2013 in The first Chair and Department of Cardiology and Cardiology Outpatient Clinic at the Medical University of Warsaw. The study was conducted in accordance with the Declaration of Helsinki. Informed consent was obtained from each patient prior to enrolment to the study. All the participants of the study gave permission to the investigators to use obtained data for scientific purposes.

The analyzed database included 230 patients with a history of syncope. Patients with heart and brain diseases were excluded and only those suffering from neuro-cardiogenic syncope were considered eligible for further investigation. The final study group consisted of 80 patients. All participants underwent a HUTT for diagnosis of VVS, according to the Westminster Protocol [30]. For Vasovagal syncope (VVS) classification the Vasovagal Syncope International Study (VASIS) criteria were applied [42]. In the passive phase of the test, 57 patients experienced syncope and we signed them as HUTT(+) group. Among the remaining 23 patients the passive phase of the test was uneventful, and they developed syncope only after pharmacological provocation with nitroglycerine. This group was described as HUTT(−). There were 17 women (aged 20–62 years, with a mean of 32.3 ± 12 years) and 6 men (aged 22–56 years, with a mean of 43 ± 15 years) in the HUTT(−) group. The HUTT(+) group consisted of 43 women (aged 18–66 years, with a mean of 35.6 ± 16 years) and 14 men (aged 18–59 years, with a mean of 41.7 ± 15.6 years). The baseline characteristics of the study group was presented in the Table 1. The research was approved by The Ethics Committee of The Medical University of Warsaw, Poland (approval number: AKBE/51/2018).

### 3.2. Head up Tilt Test

The head up tilt test (HUTT) is the main diagnostic procedure in patient assessment for vasovagal syndrome. In our Cardiology Outpatient Clinic the test is performed with The Task Force Monitor (TFM) system device (CNSystem, Graz, Austria). This device is used for the assessment of neuro-cardiogenic syncope [32,33,34]. It is composed of two parts: a lift table with a footboard and abdominal straps and devices for continuous monitoring of ECG, ICG and blood pressure. During the test, the patient lies on the table that can be tilted to different angles within a range of 60 to 90 degrees. All patients were fasting prior to the test and they restrained from consumption of coffee and alcohol the day before the test. The patients taking betablockers stopped taking drugs two days before the test. The patients taking others antihypertensive drugs have continued the treatment despite carrying out the tilt test. The tests were performed in the morning in a quiet, dimmed-light room with controlled temperature of 23–24 °C. The procedure was performed according to the modified Westminster protocol [30]: 20-min rest in the supine position followed by rapid (within 5 s) tilt to 60 degrees and remaining upright for 45 min or until syncope or pre-syncope occurred. Otherwise, the test was prolonged by additional 20 min while the patient was administered 0.4 mg nitroglycerine (NTG) sublingually in order to provoke syncope [43].

For three patients who did not experience syncope after the initial 45 min, but had contraindications to NTG administration due to a significant drop in the sBP (below 100 mmHg at the end of the passive phase) the test was prolonged by another 15 min, resulting in occurrence of syncopy in each case.

The time range from the tilt to syncope in the passive phase of the test was 3–52 min with an average of 20 min. In cases with pharmacological provocation, syncope occurred in a time range of 2–6.8 min, with an average of 3.8 min after the administration of NTG.

### 3.3. Data Analysis and Statistical Methods

As mentioned above, during HUT testing three physiological biosignals were recorded: high resolution ECG (2-channels with a sampling frequency of 1000 Hz), sBP and dBP measured continuously and ICG. The analysis was based on: RRI extracted from the ECG recordings, SV determined from the ICG curve and blood pressure (sBP, dBP).

**Figure 1:**
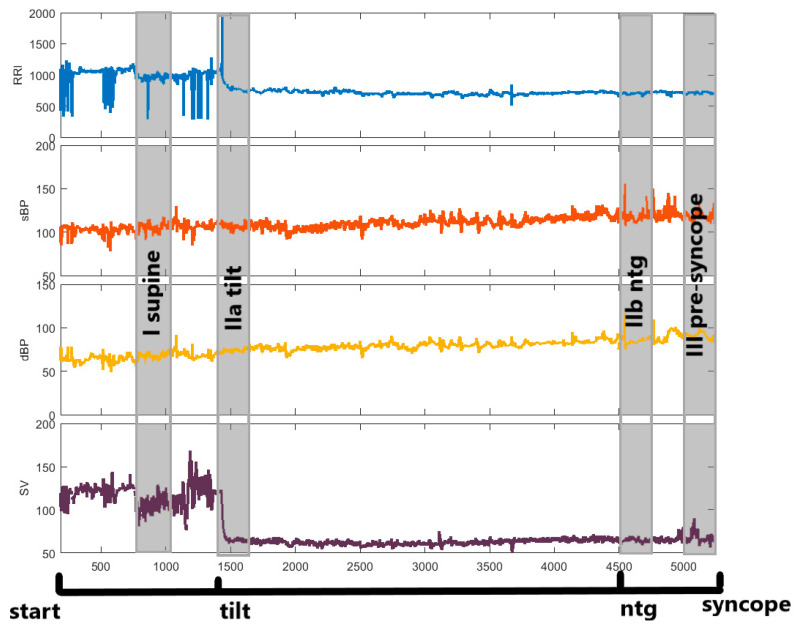
Scheme of consecutive HUTT phases. Portions of recorded data used for entropy calculations are marked as grey zones. Phase IIb (ntg) was performed only in the HUTT(−) group. Abbreviations: HUTT—head up tilt test; HUTT(−)—patients without syncope during the passive phase of HUTT, requiring pharmacological provocation with nitroglycerine (ntg).

The TFM system recorded the signals on a beat-to-beat basis. All the signals were verified by a clinician. The ectopic values were excluded and they did not exceed 5%. The analysis was performed on raw data and the signals were not detrended. Figure 1 presents a scheme of the measurements during the HUTT. In each stage of the HUTT (supine, tilt, NTG, pre-syncope) we selected four 250-beats intervals of data, called “windows”, and respectively labelled: phase I, phase IIa, phase IIb and phase III. The windows are marked as grey zones.

The entropy measures (Sample Entropy, Fuzzy Entropy, Shannon Entropy, Conditional Entropy and Permutation Entropy) were determined for RRI, sBP, dBP and SV in each window, separately for the HUTT(+) and HUTT(−) groups. Descriptive statistics of the entropies were presented as a mean ± std. Due of lack of normal distribution and variance equality, statistical analyses were performed with nonparametric tests. Comparisons of the entropies between the windows were performed with the Friedman test and the post-hoc multicomparison test. For comparisons of the entropies between the HUTT(+) and HUTT(−) groups within each HUTT phase, the Mann-Whitney test was used. For variables showing significant differences between both groups (i.e., HUTT (+) vs. HUTT(−)) in supine position, ROC analysis was additionally performed. The significance level was α = 0.05. Statistical calculations were performed with Matlab 2017b and Statistica 13 software. For entropy calculations, algorithms from Physionet [44] and J. Monge-Alvarez (available on Mathworks website) [45] were employed. The parameters applied for entropy measures calculations are presented in Table 2. To choose the input parameters we followed the recommendation of the literature. In the slow signals the meaningful results were found for embedding dimension *m* = 2. The choice of *r* was widely discussed in the literature and r=0.2·SD was found as optimal value in cardiological researches. Choosing of too large *r* will result in loosing of essential features of the signal; however too small value of *r* will result in too big impact of the noise on the complexity measure.

## 4. Results

### 4.1. Comparisons of Entropies between HUTT Phases in the HUTT(+) and HUTT(−) Group

**Figure 2:**
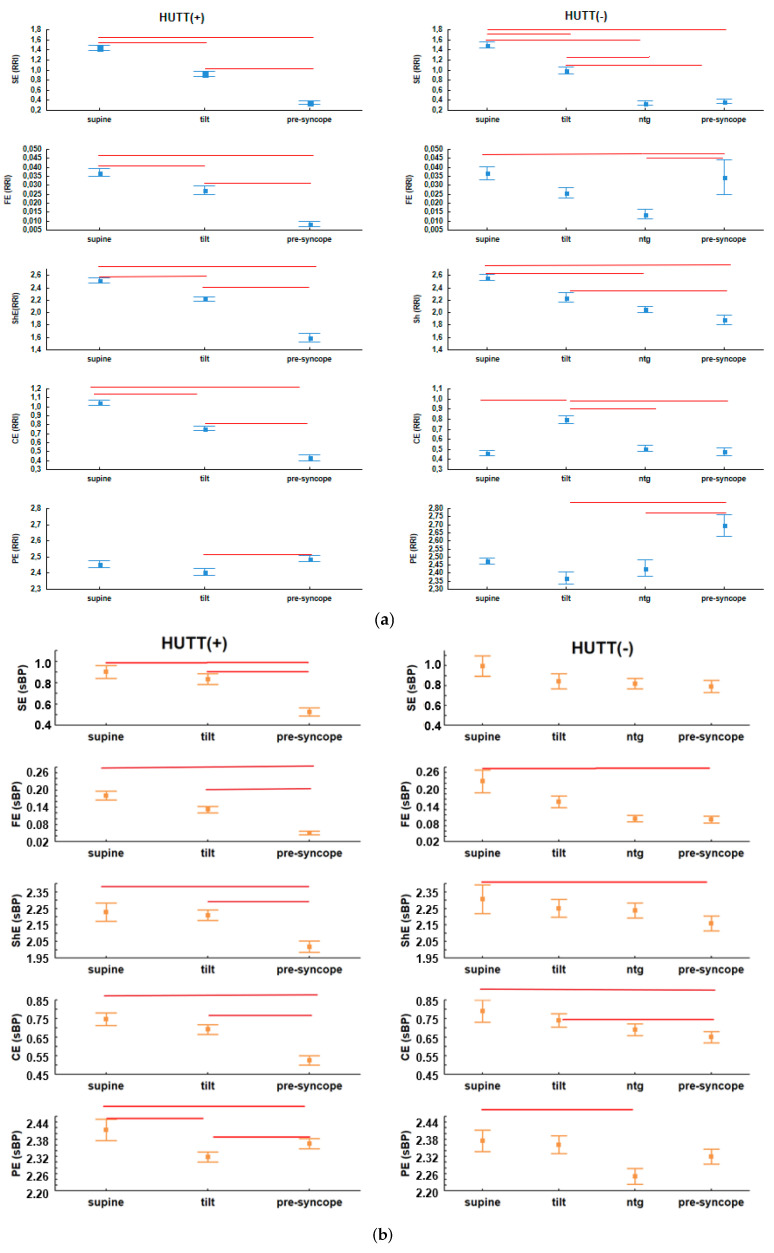

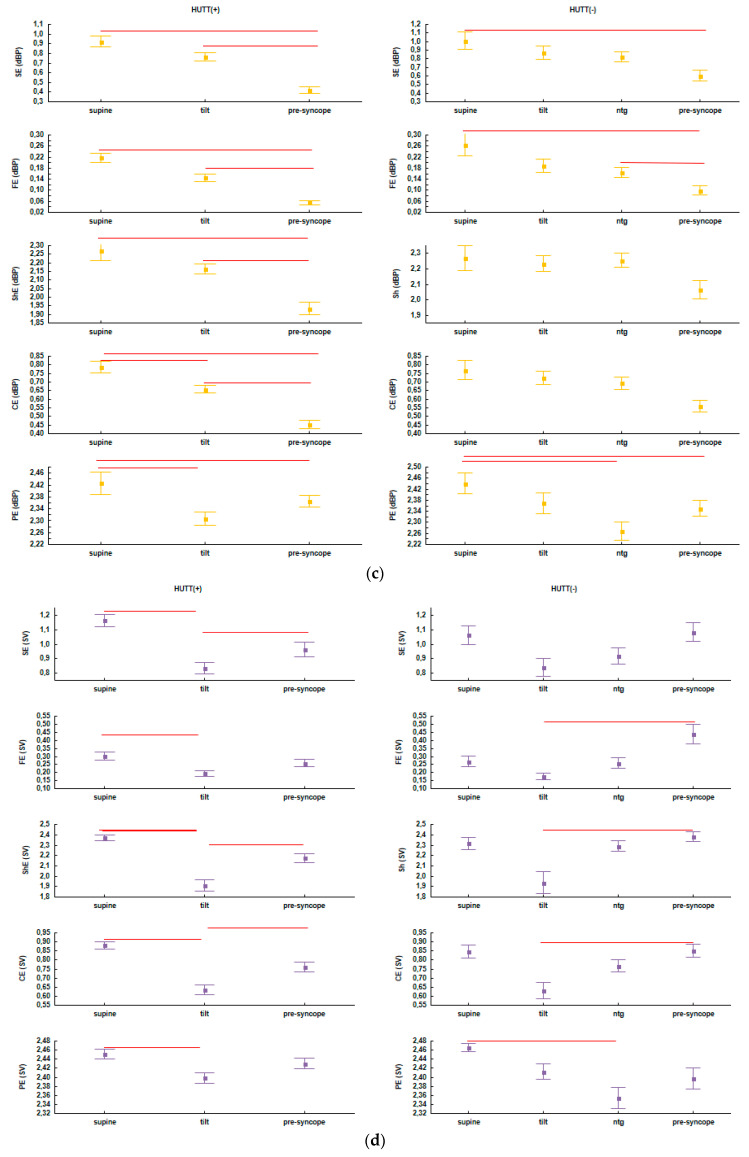
The entropies: Sample Entropy (SE) [29], Fuzzy Entropy (FE), Shannon Entropy (ShE), Conditional Entropy (CE) and Permutation Entropy (PE) in consecutive HUTT phases for the HUTT(+) (on the left) and HUTT(−) group (on the right) for: (**a**) RRI, (**b**) sBP, (**c**) dBP, (**d**) SV. Abbreviations: dBP—diastolic blood pressure, HUTT—head up tilt test, HUTT(−)—patients without syncope during the passive phase of HUTT, requiring pharmacological provocation with nitroglycerine (ntg), HUTT(+)—patients with syncope during the passive phase of HUTT, RRI—R-R intervals, sBP—systolic blood pressure, SV—stroke volume.

As described above, a set of entropies (SE, FE, ShE, CE and PE) was calculated for the parameters recorded during consecutive HUTT phases (Figure 1). Table 3 and Table 4 present descriptive statistics for the HUTT(+) and HUTT(−) groups, respectively. The statistics are presented as a mean ± standard deviation (std). Figure 2 (panels a, b, c and d) shows plots of the entropies separately for each group. The plots of entropies calculated for RRI are marked in blue, for sBP—in orange, for dBP—in yellow and for SV—in purple. The squares in the plots represent means and the whiskers—standard error (SEM). Significant results of multicomparison tests are marked as red lines.

### 4.2. Comparisions of Entropies in the HUTT(+) versus HUTT(−) Group in Individual Phases of HUTT

We performed comparisons of the entropies between both groups of patients in 3 phases of the test common for both groups (i.e., phase I, IIa and III), using the Mann–Whitney test. The results are presented in Table 5.

**Figure 3:**
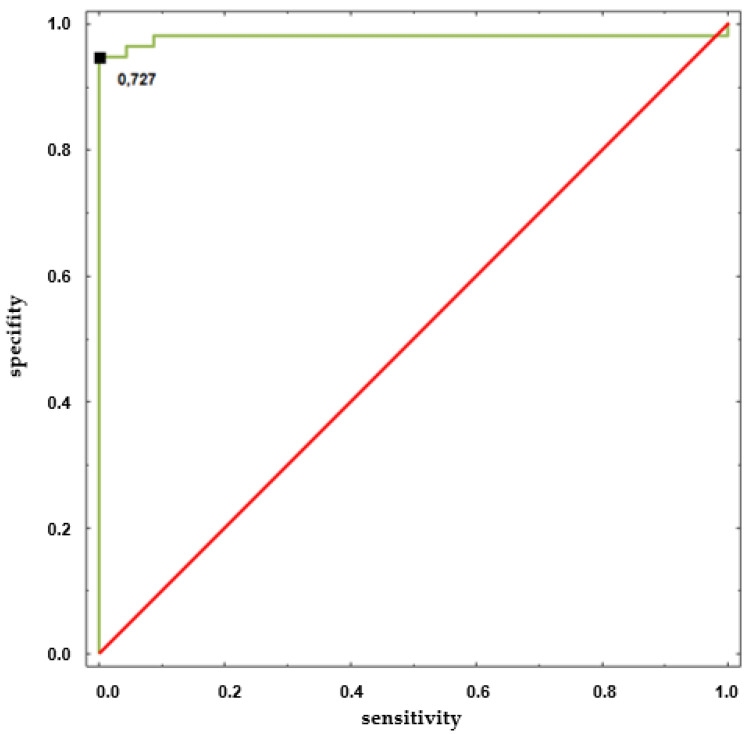
ROC curve of CE(RRI) in phase I for prediction of a positive result of the passive phase of HUTT. The threshold value is 0.727, AUC = 0.98 (CI: 0.94–1), *p* = 0.0001.

We found that the value of CE(RRI) in the HUTT(−) group in the supine position was significantly lower than in the HUTT(+) group. To define the threshold value of CE (RRI) for prediction of a positive outcome of the passive phase of HUTT, we performed ROC analysis, results of which are presented in Figure 3.

## 5. Discussion

The best diagnostic tests have some fundamental features such as high sensitivity and specificity, easiness of performance and feasibility of clinical application. It is also commonly sought to be noninvasive and cause no or only minimal distress to the patient. The HUTT is a noninvasive and painless diagnostic procedure for diagnosing syncope, but is it uncomfortable and lengthy. The possibility of prediction of occurrence of vasovagal syncope based on measurements in supine position or in response to tilt would shorten the test duration from about 1–1.5 h to 25 min. A possibility of quick prediction of the outcome of the passive phase of HUTT would also allow to omit this phase and proceed to pharmacological provocation straight after the tilt.

The idea of looking for such a predictor was also considered by other researchers [46,47,48,49,50,51]. The criterion for a negative HUTT outcome based on heart rate (HR) analysis during 6 min following an upright tilt was proposed by Mallat et al. [50]. They showed that a slight increase of HR (≤18 bpm) in the first 6 min following the tilt predicts a negative HUTT result [50]. In turn, Sutton et al. observed oscillations in blood pressure during HUTT in its passive phase and after provocation with ntg in patients who had experienced syncope in test with isopropanol [48]. HRV has also been investigated as a possible predictor of HUTT outcome, but the results are rather inconclusive. Some authors suggest that there are no significant differences between patients with positive and negative HUTT outcomes [47,52,53,54]. However, there are observations indicating for a higher sympathetic [46,55] and parasympathetic activity [46,47,55] in patients with VVS as compared with healthy subjects. Comparing patients who experienced syncope in the passive phase with those who required provocation with ntg, Erfemof et al. observed a decrement in the parasympathetic activity in patients with a positive response to ntg-provocation [56]. The investigations mentioned above were based solely on separate analysis of RRI, sBP and dBP. In our previous papers [28,29], we also analyzed the short-time response to tilt and compared RRI, sBP, dBP and SV in the supine and tilted position. In this paper, we focus on determining which kind of entropies is suitable for successful prediction of HUTT outcome based on the response to tilt. We also made an attempt to differentiate the patients who developed syncope during the passive phase of HUTT from those who required NTG provocation. The application of entropy measures to assess changes in heart rate dynamics has been extensively investigated [1,2,3,4,5,6,7,8,9,10,17,20,21,22,23,24,40]. Wejer et al. [20] measured the complexity of cardiovascular regulation during tilt test in patients suffered from VVS and healthy subject using Shannon Entropy. They noticed statistically higher entropy of sBP for patients suffering from VVS than for healthy subject in the supine position. However, in response to the tilt, this relation switches. We did not observed the significant differences between ShE (SBP) measured for HUTT(−) and HUTT(+) groups (Table 3). The mentioned authors also noticed significant decrease of ShE(RRI) in response to the tilt in both study group. They concluded, that in case of VVS patients, the reduction of entropy was stronger than for healthy subjects. We also observed significant decrease of ShE(RRI) in response to the tilt for HUTT(+) group. In case of HUTT(−) the decrease of ShE(RRI) (phase I vs. IIa) was not significant (Figure 1a). Such observations are similar to the investigation performed by Reulecke et al. [21]. Those authors suggested that a loss of complexity of RRI is directly connected with disease. In the analysis of complexity of RRI and blood pressure conducted on women suffering from VVS Reulecke et al. [24] also observed significant decreasing of entropy measure of RRI in response to the tilt. The mentioned observations are compatible with decreasing of HRV and thus with increased sympathetic activity in response to the tilt. Nonetheless, the authors noticed significant increasing of entropy of sBP in response to the tilt. Our analysis does not confirm that result. In fact, we did not observed significant differences of the entropy measures of sBP due to the orthostatic stress, but we observed significant decreasing of CE(dBP) and PE(dBP) in response to the tilt only for HUTT(+) group. Tseng Li et al. [17] found significant differences in response to the tilt between complexity index of blood pressure calculated for VVS patients and a healthy subject. They concluded that autonomic nervous system (ANS) of VVS patients reacts to the lesser extent to the orthostatic stress than autonomic nervous system of a healthy subject. In effect, VVS patients have lower adaptability under the stimulus of ANS by orthostatic stress.

In a population of patients with VVS Graff et al. demonstrated that in baseline recordings of RRI display significant differences in SE and PE between patients with a positive and negative HUTT [19]. However, when comparing patients with the same reaction to HUTT, but different breathing patterns (i.e., spontaneous vs. controlled), they did not observe significant differences [27]. Porta et al. [57] reported a decrease in the entropy of RRI during HUTT. They concluded that due to dominance of the sympathetic tone, the regularity of RRI increased and in consequence the complexity was lower. In our study, group, we also observed among HUTT(+) patients a significant decrease in all, except for PE(RRI), of the investigated entropy measures (SE (RRI) (*p* = 0.00001), FE(RRI) (*p* = 0.0022), ShE(RRI) (*p* = 0.00002), CE(RRI) (*p* < 0.00001)) in response to tilt (phase I vs. IIa). However in the HUTT(−) group only SE(RRI) was significantly higher in phase I than in phase IIa. Conversely, CE(RRI) (*p* = 0.0001) was significantly higher in phase IIa than in phase I. Comparisons between the tilt and pre-syncope phase (IIa vs. III) showed significantly lower values for the majority of entropy measures in phase III in the HUTT(+) group: SE(RRI) (*p* = 0.00001), FE(RRI) (*p* < 0.0001), ShE(RRI) (*p* = 0.002), CE(RRI) (*p* = 0.00076), while PE(RRI) was significantly higher in phase III not only in the HUTT(+) group (*p* = 0.0395), but also in HUTT(−) patients (*p* = 0.0006) (Figure 2a).

Investigating the response of sBP to tilt (phase I vs. phase IIa), we found that the only measure of entropy which displayed significant changes was PE(sBP) (*p* = 0.00001) measured in HUTT(+) patients. We found no significant changes in the entropy measures describing sBP response to tilt for HUTT(−) patients. Occurrence of syncope is always preceded by a drop in blood pressure. We observed no concomitant drop of complexity of sBP in the HUTT(−) group in phase III vs. phase IIa. In the HUTT(+) group however, only PE(sBP) was significantly higher in phase III than in phase IIa (*p* = 0.0395), while the other entropies were significantly lower (SE(sBP) (*p* = 0.00011), FE(sBP) (*p* < 0.0001), ShE(sBP) (*p* = 0.001), CE(sBP) (*p* < 0.00001)) (Figure 2b).

For dBP, we observed decreasing complexity in response to tilt (phase I vs. phase IIa) in the HUTT(+) group for CE(dBP) (*p* = 0.0237) and PE(dBP) (*p* = 0.0001). In HUTT(−) patients we found no significant differences between the analyzed entropies in response to tilt. In the latter group a significant drop of FE(dBP) in the pre-syncope phase (phase IIb vs. phase III: *p* = 0.0223) was present. In the HUTT(+) group the following measures of entropy were significantly lower in the pre-syncope phase: FE(dPB) (*p* < 0.00001), Sh(dBP) (*p* = 0.00036) and CE(dBP) (*p* = 0.00005).

As mentioned in the introduction, the measurement of SV for diagnosing syncope is not officially recommended and the values of SV recorded during HUTT are not taken into consideration in the classification of VVS. According to our research, in HUTT(+) patients all measures of entropy investigated for SV demonstrated a significant drop of entropy in phase I as compared with phase IIa (SE(SV) (*p* = 0.00001), FE(SV) (*p* = 0.01), ShE(SV) (*p* = 0.00001), CE(SV) (*p* = 0.00001), PE(SV) (*p* = 0.002)). However, in the HUTT(−) no significant changes of any of the analyzed entropy measures were found in response to tilt (Figure 2d). In both groups an increase in the entropy of SV was noted in the pre-syncope phase. This increase was statistically significant only in the HUTT(+) group (Sh(SV) (*p* = 0.00025), CE(SV) (*p* = 0.004)) (Figure 2d).

The results of calculations of different types of entropy demonstrated presence of differences in the changes of irregularity of signals during HUTT in HUTT(+) as well as in HUTT(−) patients. We also compared both groups with respect to each measure of entropy in each phase of the test. In the supine position the CE(RRI) was significantly lower in the HUTT(−) than in HUTT(+) group. This measure of entropy is the only one to differentiate the two groups in the supine position (Table 5) and can be considered as a predictor of the outcome of tilt. At tilt (phase IIa) there were no differences between the signals’ complexity between both groups and, based on the analyzed entropy measures, we were not able to indicate any types of entropy which could distinguish between these two groups in phase IIa. In the pre-syncope phase, higher irregularity of RRI was observed in the HUTT(−) than in the HUTT(+) group (FE(RRI) (*p* = 0.000007) and PE(RRI) (*p* = 0.002)). The complexity of blood pressure showed significant differences between both groups. The entropy of sBP was significantly higher in HUTT(−) than in HUTT(+) patients (SE(sBP) (*p* = 0.0006), FE(sBP) (*p* = 0.000001), ShE(sBP) (*p* = 0.01), CE(sBP) (*p* = 0.003)). The entropy of dBP was also significantly higher in the HUTT(−) group, compared with the HUTT(+) group (SE(dBP) (*p* = 0.0001), FE(dBP) (*p* = 0.0007), CE(dBP) (*p* = 0.012)) (Table 5). Similarly, the entropy of SV was also higher in HUTT(−) vs. HUTT(+) patients (FE(SV) (*p* = 0.007), ShE(SV) (*p* = 0.01)) in the pre-syncope phase.

The results of our research show that the most significant differences between the HUTT(−) and the HUTT(+) group occur in the pre-syncope phase, what could possibly be an effect of nitroglycerine administration. In consequence, the irregularity of the measured signals directly prior to syncope is supposedly higher in the HUTT(−) than in the HUTT(+) group.

All entropy measures applied in our study appear to be useful for assessment of the complexity of biosignals measured during HUTT performed for diagnosing VVS. Although special attention was paid to Conditional Entropy (CE) and Fuzzy Entropy (FE), significant differences between the HUTT(−) and the HUTT(+) group in the supine position were observed only for CE(RRI). The other measures of complexity in the supine position or at tilt are not useful for prediction of HUTT outcome. The ROC analysis performed for CE(RRI) identified the threshold value of CE(RRI) at 0.727 with AUC = 0.98 (CI: 0.94–1) (Figure 3). Values of CE(RRI) higher than 0.727 indicate a positive result of the passive phase of HUTT. Although interesting and possibly of clinical relevance, this result should be confirmed and validated in larger studies addressing possible gender and age-driven differences.

In the pre-syncope phase, Fuzzy Entropy (FE) was the only measure of entropy which differed significantly in HUTT(+) vs. HUTT(−) patients. In the HUTT(−) group the complexity of signals assessed with FE was higher than in the HUTT(+) group, for all signals. Also, a higher number of significant differences in response to tilt was observed in the HUTT(+) than in the HUTT(−) group In the latter group, the complexity of the signals in response to tilt did not change as robustly as in the former.

The only parameter for which all entropy measures were significantly different in phase I, as compared with phase IIa, was SV; however this finding was true only for HUTT(+) patients. In the HUTT(−) group no significant differences between the entropy measures were found. Based on these results we suggest inclusion of SV into VVS classification and its possible clinical use as a predictor of the results of passive HUTT.

## 6. Conclusions

The entropy measures: Sample Entropy (SE), Fuzzy Entropy (FE), Shannon Entropy (ShE), Conditional Entropy (CE), Permutation Entropy are useful tools in analysis of complexity of RRI, blood pressure and SV recorded during head up tilt test. The best prediction value with the threshold of 0.727 has the Conditional Entropy (RRI) due to its statistically significant differences between HUTT(+) and HUTT(−) in supine position. In turn Fuzzy Entropy (FE) has indicated significant differences between HUTT(+) and HUTT(−) in pre-syncope phase for all recorded signals. In the HUTT(+) group all entropy measures were significantly different for SV in response to the tilt. Therefore, we suggest including the measurements of SV as a standard measurement in VVS diagnosis. The obtained results are very interesting but the analyses have some limitations.

In our study, we did not investigate the possible impact of gender and age due to a limited size of the study group. Also, instead of testing the calculation of entropy measures using various embedding dimensions, threshold parameters and time delay, we confined ourselves to analysis using parameter settings adopted in other cardiovascular investigations.

## Figures and Tables

**Table 1:** The baseline characteristics of the study group. The parameters: age, sBP, dBP and HR are presented as (mean ± std). The other parameters are number of patients.

Baseline	HUTT(+) (*n* = 57: *F* = 43, *M* = 14)	HUTT(−) (*n* = 23: *F* = 17, *M* = 6)
Female-age (year)	35.6 ± 16	32.3 ± 12
Male-age (year)	41.7 ± 15.6	43 ± 15
HR (bpm)	72.06 ± 9.98	70.46 ± 11.35
sBP (mmHg)	108.6 ± 25.4	102.6 ± 20.4
dBP(mmHg)	68.0 ± 19.7	66.1 ± 16.8
Hypertension	3	0
Diabetes	0	0
Medication	2	4

**Table 2:** Input parameters for calculation of: Sample Entropy (SE), Fuzzy Entropy (FE), Shannon Entropy (ShE), Conditional Entropy (CE) and Permutation Entropy (PE).

Entropy	Embedding Dimension (*m*)	Threshold (*r*)	Time Delay (τ)
SE	2	0.2·SD	1
FE	2	0.2·SD	1
ShE	2	-	1
CE ^1^	2	-	1
PE	3	-	1

^1^ quantization level = 6.

**Table 3:** Descriptive statistics (mean ± std) of entropies: SE, FE, ShE, CE and PE in consecutive phases (I, IIa and III) of tilt test for the HUTT(+) group. Abbreviations: CE—Conditional Entropy, dBP—diastolic blood pressure, FE—Fuzzy Entropy, HUTT(+)—patients with syncope during the passive phase of tilt test, PE—Permutation Entropy, RRI—R-R intervals, sBP—systolic blood pressure, SE—Sample Entropy, ShE—Shannon Entropy, SV—stroke volume.

Parameter	Phase I (supine)	Phase IIa (tilt)	Phase III (pre-syncope)
***Sample Entropy*** [29]			
SE (RRI)	1.35 ± 0.30	0.88 ± 0.30	0.34 ± 0.20
SE (sBP)	0.88 ± 0.44	0.82 ± 0.30	0.51 ± 0.30
SE (dBP)	0.91 ± 0.42	0.76 ± 0.31	0.41 ± 0.30
SE (SV)	1.16 ± 0.32	0.83 ± 0.30	0.95 ± 0.40
***Fuzzy Entropy***			
FE (RRI)	0.037 ± 0.017	0.027 ± 0.017	0.008 ± 0.01
FE (sBP)	0.18 ± 0.12	0.13 ± 0.083	0.05 ± 0.046
FE (dBP)	0.22 ± 0.13	0.14 ± 0.096	0.05 ± 0.06
FE (SV)	0.30 ± 0.18	0.19 ± 0.13	0.26 ± 0.17
***Shannon Entropy***			
Sh (RRI)	2.52 ± 0.30	2.22 ± 0.25	1.59 ± 0.56
Sh (sBP)	2.23 ± 0.41	2.20 ± 0.23	2.02 ± 0.25
Sh (dBP)	2.27 ± 0.42	2.16 ± 0.23	1.93 ± 0.27
Sh (SV)	2.37 ± 0.21	1.91 ± 0.43	2.17 ± 0.32
***Conditional Entropy***			
CE (RRI)	1.05 ± 0.22	0.76 ± 0.18	0.43 ± 0.26
CE (sBP)	0.75 ± 0.25	0.70 ± 0.20	0.52 ± 0.18
CE (dBP)	0.78 ± 0.26	0.66 ± 0.18	0.45 ± 0.19
CE (SV)	0.88 ± 0.15	0.64 ± 0.20	0.76 ± 0.20
***Permutation Entropy***			
PE (RRI)	2.46 ± 0.15	2.40 ± 0.16	2.49 ± 0.15
PE (sBP)	2.41 ± 0.28	2.32 ± 0.14	2.36 ± 0.14
PE (dBP)	2.43 ± 0.28	2.30 ± 0.17	2.36 ± 0.15
PE (SV)	2.45 ± 0.08	2.4 ± 0.09	2.43 ± 0.09

**Table 4:** Descriptive statistics (mean ± std) of entropies: SE, FE, ShE, CE and PE in consecutive phases (I, IIa, IIb and III) of tilt test for the HUTT(−) group. Abbreviations: CE—Conditional Entropy, dBP—diastolic blood pressure, FE—Fuzzy Entropy, HUTT(−)—patients without syncope during the passive phase of tilt test, requiring pharmacological provocation with nitroglycerine (ntg), PE—Permutation Entropy, RRI—R-R intervals, sBP—systolic blood pressure, SE—Sample Entropy, ShE—Shannon Entropy, SV—stroke volume.

Parameter	Phase I (supine)	Phase IIa (tilt)	Phase IIb (ntg)	Phase III (pre-syncope)
***Sample Entropy*** [29]				
SE (RRI)	1.50 ± 0.30	1.0 ± 0.3	0.34 ± 20	0.38 ± 0.24
SE (sBP)	1.0 ± 0.50	0.84 ± 0.36	0.81 ± 0.25	0.80 ± 0.30
SE (dBP)	1.01 ± 0.50	0.86 ± 0.36	0.82 ± 0.26	0.60 ± 0.30
SE (SV)	1.06 ± 0.30	0.84 ± 0.30	0.92 ± 0.30	1.08 ± 0.30
***Fuzzy Entropy***				
FE (RRI)	0.037 ± 0.017	0.026 ± 0.013	0.014 ± 0.012	0.034 ± 0.046
FE (sBP)	0.25 ± 0.20	0.17 ± 0.10	0.10 ± 0.05	0.10 ± 0.06
FE (dBP)	0.26 ± 0.20	0.19 ± 0.12	0.16 ± 0.85	0.10 ± 0.07
FE (SV)	0.27 ± 0.15	0.18 ± 0.10	0.26 ± 0.15	0.44 ± 0.29
***Shannon Entropy***				
Sh (RRI)	2.56 ± 0.23	2.23 ± 0.35	2.05 ± 0.25	1.88 ± 0.36
Sh (sBP)	2.30 ± 0.42	2.24 ± 0.25	2.24 ± 0.22	2.16 ± 0.22
Sh (dBP)	2.27 ± 0.39	2.24 ± 0.25	2.24 ± 0.22	2.07 ± 0.29
Sh (SV)	2.32 ± 0.28	1.92 ± 0.28	2.32 ± 0.18	2.38 ± 0.24
***Conditional Entropy***				
CE (RRI)	0.46 ± 0.13	0.80 ± 0.19	0.51 ± 0.15	0.48 ± 0.20
CE (sBP)	0.79 ± 0.29	0.74 ± 0.17	0.70 ± 0.15	0.65 ± 0.14
CE (dBP)	0.77 ± 0.27	0.72 ± 0.19	0.69 ± 0.17	0.56 ± 0.17
CE (SV)	0.85 ± 0.17	0.63 ± 0.21	0.77 ± 0.15	0.85 ± 0.17
***Permutation Entropy***				
PE (RRI)	2.47 ± 0.09	2.37 ± 0.18	2.43 ± 0.24	2.70 ± 0.32
PE (sBP)	2.37 ± 0.18	2.36 ± 0.15	2.25 ± 0.13	2.32 ± 0.13
PE (dBP)	2.44 ± 0.17	2.37 ± 0.18	2.27 ± 0.16	2.35 ± 0.13
PE (SV)	2.46 ± 0.05	2.41 ± 0.08	2.35 ± 0.11	2.40 ± 0.11

**Table 5:** Results of Mann–Whitney test for comparisons of entropies between HUTT(−) and HUTT(+) groups in phase I, IIa and III of HUTT. ↑ and ↓ respectively indicate a higher and lower value of an analysed parameter in the HUTT(−) group in comparison with the HUTT(+) group. Statistically significant results (*p* < 0.05) are highlighted in red. Abbreviations: CE—Conditional Entropy, dBP—diastolic blood pressure, FE—Fuzzy Entropy, HUTT—head up tilt test, HUTT(−)—patients without syncope during the passive phase of HUTT, requiring pharmacological provocation with nitroglycerine (ntg), HUTT(+)—patients with syncope during the passive phase of HUTT, PE—Permutation Entropy, RRI—R-R intervals, sBP—systolic blood pressure, SE—Sample Entropy, ShE—Shannon Entropy, SV—stroke volume.

Parameter	HUTT(−) vs. HUTT(+)					
	Phase I (supine)	*p*	Phase IIa (tilt)	*p*	Phase III (pre-syncope)	*p*
***Sample Entropy*** [21]						
SE (RRI)	↑	0.46	↑	0.48	↑	0.52
SE (sBP)	↑	0.43	↑	0.99	↑	0.0006
SE (dBP)	↑	0.35	↑	0.15	↑	0.0001
SE (SV)	↓	0.13	↑	0.73	↑	0.24
***Fuzzy Entropy***						
FE (RRI)	↓	0.98	↓	0.85	↑	0.000007
FE (sBP)	↑	0.30	↑	0.14	↑	0.00004
FE (dBP)	↑	0.49	↑	0.13	↑	0.0007
FE (SV)	↓	0.40	↓	0.71	↑	0.007
***Shannon Entropy***						
Sh (RRI)	↑	0.50	↑	0.57	↑	0.07
Sh (sBP)	↑	0.23	↑	0.48	↑	0.01
Sh (dBP)	↑	0.88	↑	0.24	↑	0.055
Sh (SV)	↓	0.52	↑	0.86	↑	0.01
***Conditional Entropy***						
CE (RRI)	↓	0.00001	↑	0.36	↑	0.33
CE (sBP)	↑	0.26	↑	0.29	↑	0.003
CE (dBP)	↓	0.89	↑	0.11	↑	0.012
CE (SV)	↓	0.36	↓	0.92	↑	0.09
***Permutation Entropy***						
PE (RRI)	↑	0.88	↓	0.32	↑	0.002
PE (sBP)	↓	0.12	↑	0.27	↓	0.18
PE (dBP)	↑	0.46	↑	0.26	↓	0.57
PE (SV)	↑	0.81	↑	0.58	↓	0.22
